# Dementia Caregivers’ Communication With Healthcare Providers

**DOI:** 10.1093/geroni/igaf122.3319

**Published:** 2025-12-31

**Authors:** Jinmyoung Cho, Alan Stevens, Jennifer Thorud, Jordan Reese, Marcia Ory

**Affiliations:** Saint Louis University, Saint Louis University, Missouri, United States; Baylor Scott & White Health, Belton, Texas, United States; Baylor Scott & White Research Institute, Temple, Texas, United States; Baylor Scott & White Research Institute, Temple, Texas, United States; Texas A&M University, College Station, Texas, United States

## Abstract

The scope of caregiving for people living with dementia (PLWD) includes helping with activities of daily living to managing complex medical and nursing-related tasks. Engaging in multiple tasks leads to negative caregiving experiences. As dementia progresses, interactions with healthcare systems (e.g., scheduling appointments, communication about the patient’s medical conditions) are critical to providing care for the PLWD. However, caregivers’ interaction with healthcare providers remains underexplored. This study examines caregivers’ experiences with dementia patients’ healthcare providers. Using reports from 240 caregivers of dementia patients enrolled in a clinical trial, we conducted a descriptive analysis of the frequency of caregivers’ communication with PLWD’s healthcare workers and its relationship to caregivers’ experience with providers. Results showed that frequent communication with healthcare providers is significantly associated with tasks relevant to medical care such as scheduling appointments (χ2=12.89, p<.01) and coordinating care (χ2=30.02, p<.001). Caregivers who frequently communicated with patients’ healthcare providers reported providers always or usually listened (98.9%; χ2=14.67, p<.001) and asked about their understanding of patients’ treatments (77.8%; χ2=9.69, p<.01). Caregivers with limited or no communication with healthcare professionals reported significantly less communication on whether they needed help managing patients’ care treatment (64.5%; χ2=26.21, p<.001). Caregivers who did not communicate at all reported significantly lower burden (F = 4.01, p<.05) while caregivers who did communicate a lot reported higher positive aspects of caregiving compared to their counterparts (F = 7.99, p<.001). These findings underscore the importance of structured engagement between dementia caregivers and health professionals, which could alleviate caregiver burden and enhance care patient outcomes.

